# Total and horizontal distances of the foveal stereotaxic displacement can be prognostic indicators for patients with idiopathic epiretinal membrane

**DOI:** 10.3389/fmed.2023.1109471

**Published:** 2023-03-27

**Authors:** Zhengxi Zhang, Jianbo Mao, Jimeng Lao, Nuo Chen, Xinyi Deng, Yijing Chen, Jiwei Tao, Yiqi Chen, Lijun Shen

**Affiliations:** ^1^School of Ophthalmology and Optometry, Wenzhou Medical University, Wenzhou, China; ^2^Department of Retina Center, Zhejiang Provincial People’s Hospital, Hangzhou, China; ^3^Department of Retina Center, Affiliated Eye Hospital of Wenzhou Medical University, Hangzhou, Zhejiang, China

**Keywords:** idiopathic epiretinal membrane, retina, optical coherence tomography, optical coherence tomography angiography, epiretinal membrane

## Abstract

**Introduction:**

This study aimed to examine the foveal stereo deviations in the different ectopic inner foveal layer (EIFL) stages of idiopathic epiretinal membrane (iERM) and assess its predictive utility for the baseline and postoperative best-corrected visual acuity (BCVA).

**Methods:**

Based on the calculational combination of foveal displacements in the horizontal and vertical axial optical coherence tomography (OCT) images, the foveal stereotaxic displacement was estimated through the total distance (TD, the distance from the foveal bottom to the inner edge of displaced central foveal) and horizontal distance (HD, projection of the TD in the retinal plane). The preoperative TD, HD, and other OCT- and OCT angiography (OCTA)-related indicators were obtained. The correlations between structural parameters and baseline and postoperative BCVA were evaluated through correlation and multiple linear regression analyses.

**Results:**

In patients with advanced EIFL stage, there was a significant increase in the HD, TD, baseline log of the minimum angle of resolution unit for BCVA, central macular thickness (CMT), acircularity index, and incidence of microcystic macular edema (MME; *p* < 0.05). Further, they showed a decreased foveal avascular zone (FAZ) area and perimeter (*p* < 0.001). HD, TD, CMT, MME, FAZ area, and FAZ perimeter were significantly correlated with the baseline and postoperative BCVA (*p* < 0.05). TD had the highest correlation indexic and was an individual predictor of the baseline and postoperative BCVA. Moreover, FD-300 and MME were individual predictors of postoperative BCVA.

**Discussion:**

Stereoscopic foveal deviations significantly correlated with the baseline and postoperative visual acuity. TD may be used as an independent prognostic factor for BCVA.

## 1. Introduction

Idiopathic epiretinal membrane (iERM) is a common macular disease with unknown etiology. It is characterized by abnormal fibroblast proliferation on the inner macular surface; moreover, it is commonly observed in older individuals (1, 2). The contraction characteristics of iERM can cause anatomical destruction and stratified changes, which results in visual impairment, metamorphopsia, and aniseikonia as the main symptoms (3, 4). Surgical pars plana vitrectomy (PPV) with iERM peeling is the standardized procedure for releasing retinal traction and enhancing retinal repair. However, there are some cases of insufficient anatomical and functional recovery even after successful surgery without significant complications (5–7). Therefore, it is important to predict the probability of postoperative visual recovery and to determine the timing of surgery.

There has been increasing interest in central macular thickness (CMT) and ectopic inner foveal layer (EIFL), which primarily react to retinal alterations caused by vertical iERM traction (6, 8–11). However, iERM contraction or shrinkage can cause foveal displacement. This can result in the loss of light dispersion due to vessel displacement (12), and light propagation anomalies due to muller cell deformation (13–15). Daiki et al. (16) proposed tangential displacement as a parameter for characterizing tangential morphological alterations in the outer retina. They reported that the horizontal and vertical metamorphopsia scores were positively correlated with the absolute values of the vertical and horizontal tangential displacement, respectively.

Given that foveal thickening and deviating under iERM traction is a three-dimensional problem, analysis through appropriate geometry methods is necessary. To further elucidate the pathogenesis of visual impairment, we calculated the horizontal distance (HD) and total distance (TD) in iERM using horizontal and vertical B-scan images from the bottom of the central fovea to the innermost vertex of the central fovea. Further, we analyzed the correlations of HD and TD with baseline and postoperative visual acuities.

## 2. Materials and methods

### 2.1. Study participants

This retrospective study included patients diagnosed with iERM between November 2018 and March 2021 at Eye Hospital of Wenzhou Medical University. The iERM was defined as the pathologic fibrocellular membrane that lies immediately superjacent to the inner surface of the retina, excluding cause of secondary epiretinal membrane (4). Inclusion criteria were (1) aged ≥ 45 years; (2) eyes with surgery underwent the 23-gauge standard 3-port PPV that included peeling of the ERM and internal limiting membrane (ILM) and combined cataract surgery. The exclusion criteria were as follows: (1) any other intraocular disease, including diabetic retinopathy, age-related macular degeneration, or hypertensive retinopathy; (2) any history of vitreoretinal surgery; (3) axial length > 26 mm or the presence of myopia of > −6 diopters; and (4) media opacities that prevented good fundus visualization. The study procedures conformed to the tenets of the Declaration of Helsinki (Research Ethics Approval Code: 2019168K160).

### 2.2. Surgical procedures

Using 0.025% indocyanine green dye staining and a minimal intraoperative exposure time, the iERM was peeled as much as possible and internal limiting membrane were removed from an area around the macula *circa* 3–4-disc diameter large. Cases without cataract surgery or followed up for less than 11 months would be excluded from the postoperative analysis. Baseline data were obtained from all the initial participants to rule out the presence of selection bias.

### 2.3. Examination

**Figure 1:**
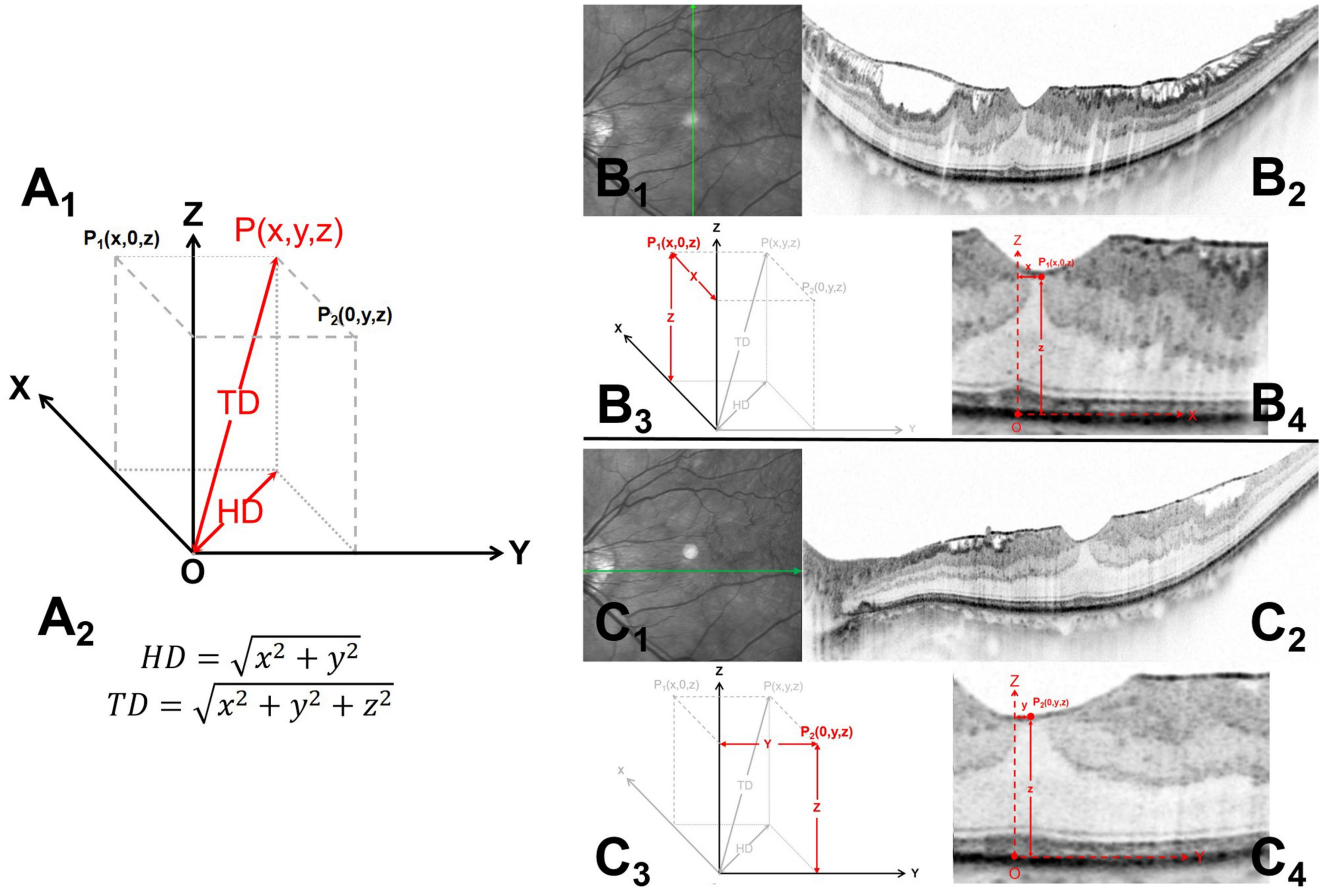
Measurements of the horizontal distance (HD) and total distance (TD) of the foveal stereo deviations on the B-scan images. A_l_ and A_2_ illustrate the schematic diagram and formula (*P_l_* and *P_2_*) for the TD and HD of the foveal stereo deviations obtained from the horizontal and vertical axial B-scan images. B_1-4_ and C_1-4_ demonstrate the methods for obtaining *P_l_* and *P_2_*, respectively. B_l_ and C_l_ indicate the scanning directions, while B_2_ and C_2_ show the complete B-scan images. B_3_ and C_3_ display the coordinate diagram corresponding to foveal deviation in the horizontal and vertical planes, and B_4_ and C_4_ provide specific annotations of *O*, *P1*, and *P2* in the B-scan images. The specific measurement methods are described below: (1) Microstructures were consistently located at the bottom of the central fovea, including the ellipsoid zone bulge, cotton ball sign (CB), foveolar detachment, and acquired vitelliform lesion. Fixation point locations were also comprehensively considered to identified central fovea. The point on the retinal pigment epithelium (RPE) at the central fovea was identified as the reference starting point (B_4_, C_4_). Vertical (B_1-2_) and horizontal (C_1-2_) axes scans centered at the coordinate origin (O) were performed. (2) Endpoint (*P*_1_, *P*_2_) refers to the innermost foveal edge at the thinnest position of the inner retina [from the inner nuclear layer (INL) to the retinal nerve fiber layer] (B_4_, C_4_). (3) A coordinate system on the RPE surface was established; moreover, the stereotaxic estimate of the offset central foveal (*P*_(x, y, z)_) can be obtained using the corresponding coordinates of the two orthogonal planes (A_1_). HD = $\sqrt{x^{2}+y^{2}}$, and TD = $\sqrt{x^{2}+y^{2}+z^{2}}$.

All patients underwent Spectralis spectral domain–optical coherence tomography (SD-OCT; Heidelberg Engineering GmbH, Heidelberg, Germany) at baseline. Single line B-scans in the horizontal and vertical axial were performed centered at the bottom of the central fovea (Figure 1). Based on the OCT measurements, we calculated the following indexes to determine the foveal stereotaxic displacement. TD reflected the distance from the foveal bottom to the displaced central foveal edge, HD was projection of the TD in the retinal plane and reflected the offset distances in the horizontal plane.

Microstructures were consistently located at the bottom of the central fovea, (17–24), including the ellipsoid zone bulge, cotton ball sign (CB), foveolar detachment, and acquired vitelliform lesion. Fixation point locations were also comprehensively considered to identified central fovea (16). The lowest point on the retinal pigment epithelium (RPE) at the central fovea was identified as the coordinate origin (O) by referring to these locations. At the fovea, the endpoints in both orthogonal B-scan images, P_1_ and P_2_, referred to the innermost foveal edge at the thinnest inner retinal position [from the inner nuclear layer (INL) to the retinal nerve fiber layer].

**Figure 2:**
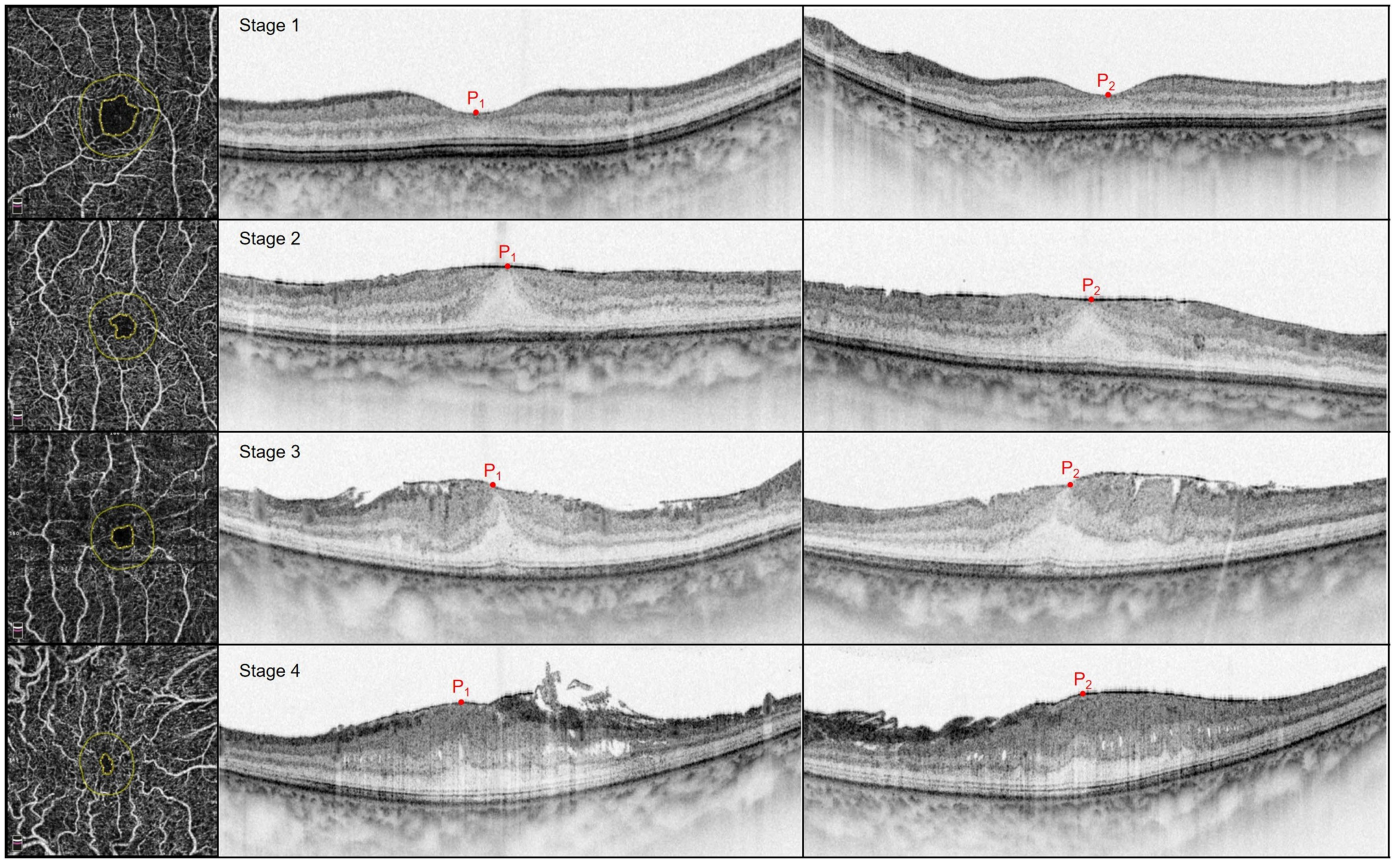
OCTA (left), vertical and horizontal axis OCT (middle and right) scans of epiretinal membranes; from top to bottom are Stages 1–4. P_1_ and P_2_ represented the foveal deviation endpoint on the vertical and horizontal axis scans, respectively. Stage 1: Mild with negligible morphologic or anatomic disruption, HD is 88 μm and TD is 213 μm. Stage 2: Loss of foveal depression without EIFL, HD is 100 μm and TD is 356 μm. Stage 3: Presence of continuous EIFL across the central fovea, HD is 405 μm and TD is 605 μm. Stage 4: Continuous EIFL with anatomic disruption, HD is 446 μm and TD is 714 μm.

A coordinate system was established as follows (Figure 2): the X and Y axis formed the RPE horizontal section, while the Z axis was the intersection line of the two orthogonal B-scan images. Both orthogonal image planes could be considered the X-Z plane and Y-Z planes, while P1 and P2 could be expressed as (x, 0, z) and (0, y, z), respectively, in coordinates. The values x, y, and z were the values projected of P_1_ and P_2_ onto different axes X, Y, and Z. P_1_ (x, 0, z) and P_2_ (0, y, z) were planar projections of P (x, y, z), which is the stereotaxic estimate of the offset central foveal. Based on the Pythagorean theorem, HD and TD could be obtained as follow: HD = $\sqrt{x^{2}+y^{2}}$ and TD = $\sqrt{x^{2}+y^{2}+z^{2}}$. Images were reviewed by two independent readers (L.S. and J.M.), who were blinded to the clinical information. The average of both measurements was used for analysis; moreover, in case of between-observer differences exceeding 5%, the observers discussed and repeated measurements.

The EIFL was observed using SD-OCT. Continuous hyporeflective and hyperreflective bands extended from the INL and inner plexiform layer to the central fovea. Further, iERM was classified into four stages (6). Stage 1 was indicated by mild with negligible morphologic or anatomic disruption. Stage 2 involved loss of foveal depression without EIFL. Stage 3 involved the presence of a continuous EIFL across the central fovea. Stage 4 was indicated by a continuous EIFL and anatomic macular disruption (Figure 2). Additionally, OCT was used to detect CB and microcystic macular edema (MME): CB was indicated by a highly reflective area between the photoreceptor inner segment and the outer segment junction line (18). MME was indicated by a lacunar hyporeflective area with a clear boundary predominantly located in the INL (25). OCT angiography (OCTA; Optovue RTVue XR, Optovue Inc., CA, United States) indicators were obtained at baseline. Further, the following foveal avascular zone (FAZ) parameters were collected: FAZ area, FAZ perimeter, acircularity index (AI), and the foveal vessel density (FD) within a 300-μm region around the FAZ. Best-corrected visual acuity (BCVA) was measured at baseline and postoperative follow-up using the Chinese standard logarithm visual chart, followed by conversion into the log of the minimum angle of resolution (logMAR) unit.

### 2.4. Statistical analysis

All statistical analyses were performed using SPSS version 23.0 (SPSS, IL, United States). Data are expressed as mean ± standard deviation. Intra- and inter-observer reliabilities were performed using intraclass correlation coefficient. Analysis of variance (ANOVA) was used for among-group comparisons. Categorical data were analyzed using the chi-square test or Fisher exact test. Between-group comparisons were performed using independent-sample and paired-sample *t* tests. The Kruskal–Wallis nonparametric and Wilcoxon signed-rank tests were used to analyze data with non-normal distribution. Correlations were analyzed through Spearman’s rank correlation. A strong, moderate, and strong correlation was indicated by 0.5 < |r| ≤ 1, 0.3 < |r| ≤ 0.5, and |r| ≤ 0.3, respectively (26). Variables with a value of *p* < 0.2 in the correlation analysis were included in stepwise multiple linear regression analyses to identify predictive factors for baseline and postoperative visual outcomes. Statistical significance was set at *p* < 0.05.

## 3. Results

### 3.1. Baseline characteristics

We included 160 eyes from 160 patients, and they were all included in the baseline statistical study to avoid selection bias. Stages 1, 2, 3, and 4 of the EIFL were detected in 40, 33, 66, and 21 eyes, respectively. There were no significant among-stage differences in age and sex ratio (*p* = 0.371, 0.209; Table 1).

**Table 1:** Characteristics of the included patients with EIFL classification.

	All	Stage 1	Stage 2	Stage 3	Stage 4	*F*	*p*
**Totality**							
Number of eyes	160	40	33	66	21		
Age	62.56 ± 9.77	60.35 ± 10.16	63.70 ± 8.81	62.80 ± 9.95	64.24 ± 9.80	1.05	0.371^†^
Male sex, *n* (%)	54 (33.8)	19 (47.5)	10 (30.3)	19 (28.8)	6 (28.6)		0.209^+^
**Follow-up completed**							
Number of eyes	91	0	23	53	15		
Age	64.10 ± 9.60	-	63.82 ± 8.39	63.55 ± 9.98	66.40 ± 10.49	0.52	0.597^†^
Male sex, *n* (%)	23 (25.3)	-	8 (34.8)	13 (24.5)	2 (13.3)		0.325^+^
Follow-up time (months)	12.08 ± 0.69	-	12.00 ± 0.67	12.13 ± 0.74	12.00 ± 0.54	0.40	0.669^†^

† One-way ANOVA test.

+ Chi-square test or Fisher exact test.

We included 91 eyes who underwent 23G PPV with combined cataract surgery and had a follow-up period of ≥11 months. We excluded three cases without cataract surgery, 13 with a short follow-up period, and 51 did not undergo surgical intervention due to minor symptoms or refusal of surgical treatment. Further, 91 eyes were operated on and had a postoperative follow-up period of 12.08 ± 0.69 months. Stages 1, 2, 3, and 4 of the EIFL were detected in 0, 23, 53, and 15 eyes, respectively. There were no significant among-stage differences in age, sex ratio, or follow-up period (*p* = 0. 597, 0. 325, 0. 669; Table 1).

### 3.2. Baseline measurements at different stages

The EIFL stage was positively correlated with the CMT, AI, and incidence of MME, as well as negatively correlated with the BCVA, FAZ area and FAZ perimeter values (all < 0.05). The EIFL stage was not correlated with the FD or CB (*p* = 0.684, 0.099; Table 2).

**Table 2:** Preoperative measurements with EIFL classification.

	Stage 1	Stage 2	Stage 3	Stage 4	*F/Z*	*p*
LogMAR BCVA	0.10 ± 0.13	0.30 ± 0.30	0.47 ± 0.27	0.74 ± 0.32	32.1	<0.001^†^
MME	3/40	4/33	9/66	9/21		0.002^+^
CB	5/40	12/33	20/66	5/21		0.099^+^
CMT, μm	316.9 ± 70.1	444.5 ± 89.7	506.3 ± 88.5	655.6 ± 137.2	102.0	<0.001*
HD, μm	45.1 ± 86.2	138.9 ± 159.5	232.9 ± 181.0	542.1 ± 327.4	74.8	<0.001*
TD, μm	251.7 ± 91.2	454.2 ± 140.5	547.9 ± 151.3	856.4 ± 281.4	101.6	<0.001*
**FAZ parameters**						
FAZ area, mm^2^	0.13 ± 0.08	0.10 ± 0.04	0.06 ± 0.06	0.04 ± 0.03	74.8	<0.001*
FAZ perimeter, mm	1.34 ± 0.49	1.28 ± 0.30	1.03 ± 0.42	0.85 ± 0.35	26.1	<0.001*
AI	1.13 ± 0.06	1.20 ± 0.10	1.23 ± 0.19	1.38 ± 0.39	9.4	0.024*
FD, %	46.53 ± 8.36	45.44 ± 4.63	45.74 ± 6.05	45.86 ± 6.69	2.1	0.684*

AI, acircularity index; BCVA, best-corrected visual acuity; CB, cotton ball sign; CMT, central macular thickness; FAZ, foveal avascular zone; FD, foveal vessel density in a 300-μm-wide region around the FAZ; HD, horizontal displacement; MME, microcystic macular edema; TD, total distance. LogMAR BCVA was used in all statistical analyses.

† One-way ANOVA test.

* Kruskal–Wallis nonparametric ANOVA test.

+ Chi-square test or Fisher exact test value.

In the pairwise comparisons, there were significant between-stage differences in the CMT and BCVA (all *p* < 0.05), as well as the FAZ area except for between Stage 3 and 4 (*p* = 0.127). MME was more common in Stage 4 than in the other stages (all *p* < 0.05). AI was lower in Stage 1 than in Stage 4 (*p* = 0.015). The FAZ perimeter significantly differed between Stages 2 and 3, Stages 1 and 4, and Stages 2 and 4 (all *p* < 0.005).

The measurements of HD and TD showed high intra- and inter-observer reliabilities (All ICC ≥ 0.940, Supplementary Table 1). The EIFL stage was significantly positively correlated with HD and TD (all *p* < 0.001). For pairwise comparisons, there were significant between-stage differences in the HD and TD (all *p* < 0.05).

When components of both orthogonal planes were compared, there was no significant difference in the HD (*p* = 0.193) or TD (*p* = 0.488).

### 3.3. Correlation analyses of visual impairment and prognosis

The baseline BCVA was negatively correlated with CMT, MME, HD, and TD, as well as positively correlated factors with the FAZ area and perimeter (Table 3). In particular, CMT, FAZ area, HD, and TD were strongly correlated with baseline BCVA. Among all metrics, the highest correlation was observed for TD. TD and MME showed a 45.4% contribution to the baseline BCVA (*F* = 51.66, *p* < 0.001); further, they were considered independent predictors (*B* = 0.539, *p* < 0.001; *B* = 0.266, *p* < 0.001).

**Table 3:** Correlation analysis of preoperative indicators with baseline logMAR BCVA.

	*r*	*p*
MME	0.416	<0.001
CB	0.145	0.067
CMT	0.750	<0.001
HD	0.654	<0.001
TD	0.766	<0.001
**FAZ parameters**		
FAZ area	−0.679	<0.001
FAZ perimeter	−0.420	<0.001
AI	0.170	0.059
FD	−0.121	0.180

AI, acircularity index; BCVA, best-corrected visual acuity; CB, cotton ball sign; CMT, central macular thickness; FAZ, foveal avascular zone; FD, foveal vessel density in a 300-μm-wide region around the FAZ; HD, horizontal displacement; TD, total distance; MME, microcystic macular edema. LogMAR BCVA and Spearman rank correlation were used in all statistical analyses.

Preoperative HD and TD were negatively correlated with the postoperative BCVA (Table 4). Moreover, the baseline MME, and CMT were negatively correlated with the last follow-up BCVA. Contrastingly, the baseline BCVA, FAZ area, FAZ perimeter, and FD were negatively correlated with the last follow-up BCVA. The baseline BCVA showed strong correlation; the CMT, HD, and TD showed moderate correlations. Among all structural parameters of the retinal, TD showed the strongest correlation.

**Table 4:** Correlation analysis of preoperative indicators with last follow-up LogMAR BCVA.

	*r*	*p*
Baseline BCVA	0.574	<0.001
MME	0.286	0.006
CB	−0.110	0.299
CMT	0.340	0.001
HD	0.362	<0.001
TD	0.406	<0.001
**FAZ parameters**		
FAZ area	−0.260	0.012
FAZ perimeter	−0.234	0.025
AI	0.116	0.272
FD	−0.255	0.015

AI, acircularity index; BCVA, best-corrected visual acuity; CB, cotton ball sign; CMT, central macular thickness; FAZ, foveal avascular zone; FD, foveal vessel density in a 300-μm-wide region around the FAZ; HD, horizontal displacement; TD, total distance; MME, microcystic macular edema. LogMAR BCVA and Spearman rank correlation were used in all statistical analyses.

Stepwise multiple linear regression analyses were conducted to identify predictive factors for the last follow-up BCVA among the baseline retinal parameters. We found that the resulting model could explain 24.3% of the variance and could significantly predict the last follow-up BCVA [*F* (11.04), *p* < 0.001]. The TD, FD, and MME significantly contributed to the model (*B* = 0.319, *p* = 0.002; *B* = −0.249, *p* = 0.008; *B* = 0.214, *p* = 0.036).

## 4. Discussion

This study had several principal findings. First, TD was an independent predictor of the baseline and postoperative BCVA in the multiple linear regression analysis. Second, the HD and TD were significantly correlated with the baseline and last follow-up BCVA, with TD showing the highest correlation coefficient among all the retinal parameters. Third, the preoperative HD and TD values significantly increased with advanced EIFL staging. There were no significant differences between the vertical and horizontal components.

Therefore, the HD and TD may accurately reflect the degree of iERM stereo traction and may be predictive factors for the degree of preoperative visual impairment and postoperative visual prognoses.

The formation of iERM shares similar characteristics as fibrosis. Specifically, the transdifferentiation of various precursor cells into myofibroblasts and the massive production of extracellular matrix proteins containing extracellular fibrils results in fibrous contraction and distortion of the normal tissue structure (4). Retinal thickening, disorganization, and altered foveal morphology can be caused by the vertical traction and horizontal tangential forces of iERM (11, 27–29). The area and depth of iERM traction were correlated with the foveal alteration and BCVA (30). Therefore, the iERM-generated forces and the resulting macular structure damage can be three-dimensionally considered. Stereo quantitative analysis of the central macular displacement might allow a better description of iERM tractions.

Although previous studies have used fundus autofluorescence imaging to evaluate retinal displacement based on relative changes in the position of blood vessels (31), this is confined to the inner retinal layer. Our study showed that the HD and TD significantly increased with staging, indicating a significant positive correlation between retinal stereo deviations with iERM severity in the whole retina. The lack of significant differences between the horizontal and vertical components of both indexes could be attributed to the indeterminate traction location, which is consistent with the results of Sakai et al. (16).

Sakai et al. (16) reported no significant correlation of the fovea’s horizontal and vertical tangential distortion with BCVA. Hsiao-Fan Tung et al. (32) reported that postoperative horizontal movement of the central fovea was positively correlated with the severity of BCVA worsening; however, this parameter cannot be preoperatively obtained. We found that both HD and TD were significantly associated with baseline BCVA, with TD showing the highest correlation index. Moreover, TD was an independent predictor of preoperative BCVA.

Ichikawa et al. (33) reported that the ratio of baseline to postoperative measurements of the distances between the intersections of retinal vessels on near-infrared spectroscopy images was correlated with the postoperative M-score. Another recent study reported that the postoperative change in the projection distance relative to the PRE layer at the vascular bifurcation was associated with BCVA at 6 postoperative months (34). However, both aforementioned parameters only reflected changes in the horizontal plane.

In our study, the last follow-up BCVA was significantly correlated with the baseline HD and TD, with TD having the highest correlation coefficient. Moreover, TD was a significant predictor of the last follow-up BCVA. Thus, serious stereo heterotopia of the retina was an adverse factor for visual prognosis. Furthermore, it is important to consider the TD when determining the timing of surgery.

The FAZ area, perimeter, and AI significantly correlated with staging. Previous studies have reported that FAZ-related parameters differed significantly compared with healthy controls (28), and changed significantly with disease severity (29, 35, 36). The FAZ area and perimeter were significantly correlated with the baseline and postoperative BCVA. The correlation between FAZ area and baseline BCVA was also proven in previous studies (28, 35, 37–39) and BCVA recovery (28, 37, 40, 41). Due to the differences in sample size, follow-up time, scanning depth range of different OCTA devices, and software vascular detection algorithm, many studies have controversial results on the correlation between FAZ-related indicators and visual acuity before (29, 35, 42, 43) and after surgery (35, 38, 43, 44). Although FD was not significantly correlated with the baseline staging, it was significantly associated with and was an independent predictor of postoperative visual acuity. Consistent with our findings, Kim et al. (41) reported that the parafoveal capillary density was significantly associated with postoperative visual acuity. This suggested that vessel displacement could alter the related tissue supply, with independent prognostic implications. Previous studies on FD-300 reported that the recovery at 6 months postoperatively did not reach statistical significance. The absence of a correlation with postoperative visual acuity was probably attributed to the limited sample size and insufficient follow-up period (8). CB was not significantly correlated with visual function, which is consistent with previous reports (22). The incidence of MME was positively correlated with EIFL staging progression; further, it was an independent predictor of the baseline and postoperative BCVA. Consistent with our findings, previous studies (45–47) have shown that MME is a nonnegligible prognostic index.

This study has several limitations. First, estimating the HD and TD through horizontal and vertical B-scan images may involve errors due to its retrospective nature. Theoretically, high-density imaging, with the 360° rotating B-scans centered on the bottom of the central fovea, can simultaneously obtain the image from the start to the endpoint. Accordingly, this may be used to accurately measure the HD and TD; however, future studies are warranted to test this hypothesis. Second, although the dose and exposure time of indocyanine green was minimized and it is relatively safe, its potential retinal toxicity still requires attention (48, 49). Third, the postoperative follow-up period was not exactly 12 months due to patient compliance issues. Forth, this was a small-scale retrospective study.

In conclusion, the baseline HD and TD were positively correlated with EIFL stage progression; moreover, they were predictors of the baseline and postoperative BCVA. TD showed the highest correlation indexes and was an independent predictor of baseline and postoperative visual acuity. FD-300 and MME, which are markers related to microvascular changes and microstructural damage, also showed significant predictive utility for postoperative visual acuity. Therefore, the HD and TD may accurately reflect the degree of iERM stereo traction and allow prediction of baseline and postoperative visual acuity.

## Data availability statement

The raw data supporting the conclusions of this article will be made available by the authors, without undue reservation.

## Ethics statement

The studies involving human participants were reviewed and approved by 2019168K160. Written informed consent for participation was not required for this study in accordance with the national legislation and the institutional requirements.

## Author contributions

LS, JM, and ZZ contributed to the conception of the study. LS, JM, ZZ, JL, NC, XD, and YijC performed the experiment. ZZ, JL, and YijC contributed significantly to analysis and manuscript preparation. ZZ, JT, and YiqC performed the data analyses and wrote the manuscript. ZZ and JM helped perform the analysis with constructive discussions. All authors contributed to the article and approved the submitted version.

## Funding

The Zhejiang Medical Health Science and Technology Project (2023KY490) and the Natural Science Foundation of Zhejiang Province (LTGY23H120005) supported this study. Nonetheless, funding bodies were not involved in the design of the study and collection, analysis, and interpretation of data and in writing the manuscript.

## Conflict of interest

The authors declare that the research was conducted in the absence of any commercial or financial relationships that could be construed as a potential conflict of interest.

## Publisher’s note

All claims expressed in this article are solely those of the authors and do not necessarily represent those of their affiliated organizations, or those of the publisher, the editors and the reviewers. Any product that may be evaluated in this article, or claim that may be made by its manufacturer, is not guaranteed or endorsed by the publisher.
