# Supplementary material for: Total and horizontal distances of the foveal stereotaxic displacement can be prognostic indicators for patients with idiopathic epiretinal membrane
Source: Front Med (Lausanne). 2023 Mar 27;10:1109471. doi: 10.3389/fmed.2023.1109471 (PMC10084726; doi:10.3389/fmed.2023.1109471)
Supplement: Supplementary file 1 [file Table_1.DOCX]

# Supplementary Table 1 The intraclass correlation coefficient analysis of horizontal distance (HD) and total distance (TD) of the foveal stereo deviations.

| Measurement | | Intra-observer I reliability | Intra-observer II reliability | Inter-observer reliability |
| --- | --- | --- | --- | --- |
|  | HD | 0.986* (0.980-0.989) | 0.984* (0.978-0.988) | 0.988* (0.984-0.991) |
|  | TD | 0.961* (0.947-0.971) | 0.948* (0.930-0.962) | 0.940* (0.919-0.956) |

The values are presented as the ICC, with 95% confidence interval, * P <0.001.
